# Health benefits package selection in oncology: a clinical audit of palliative chemotherapy for breast cancer in an Indian health insurance scheme

**DOI:** 10.1186/s12913-024-12018-4

**Published:** 2024-12-18

**Authors:** Sebastian Bauhoff, Manju Sengar, C. S. Pramesh, Anamika Dhiman, Abha Mehndiratta

**Affiliations:** 1 Department of Global Health and Population, Harvard TH Chan School of Public Health, Boston, USA; 2 Tata Memorial Hospital, Mumbai, India; 3 Center for Global Development, Washington, DC USA

**Keywords:** Clinical Audit, Hospitals, Health Insurance, India, Oncology

## Abstract

**Background:**

The effective operation of health insurance requires functioning administrative processes, including appropriate filing for reimbursements. The unlisted palliative care package is one of the most utilized oncology packages within Indian state health insurance schemes. We conducted a clinical audit to evaluate the appropriateness of claims for this package for patients with breast cancer.

**Methods:**

We obtained documentation submitted to support a random sample of insurance claims for the unlisted palliative care package for 50 patients (total) from one public and one private hospital, cataloged the available records, and assessed the appropriateness of package selection using clinical guidelines from India’s National Cancer Grid. Where sufficient documentation was available, we also assessed the clinical appropriateness of care. We also examined whether these patients received relevant care at other hospitals that participate in the scheme and, for five purposefully selected patients, whether additional documentation had been submitted alongside other claims.

**Results:**

Claims for 45 of the 50 patients had sufficient documentation to assess whether the selected package was appropriate. Among these 45 claims only 33% were selected in accordance with guidelines; the public hospital had a relatively higher share of appropriate selections. In many cases (21 claims), the palliative care package was selected for adjuvant therapy and targeted therapy. While more than half of the sampled patients had multiple related encounters and sometimes received care from multiple hospitals, reviewing additional claims did not affect our conclusions.

**Conclusion:**

Claims for the palliative chemotherapy unlisted package often had deficient documentation and were inappropriately selected.

**Supplementary Information:**

The online version contains supplementary material available at 10.1186/s12913-024-12018-4.

## Introduction

Many low and middle-income countries (LMICs) have adopted publicly funded health insurance programs to achieve universal health coverage [1–3]. As these programs become established and are being expanded, there are concerns whether the programs’ operations and quality of care may hamper efforts to improve effective coverage and, ultimately, health outcomes [4].

India has made considerable progress in improving access to key health services in the last two decades [5], partly by instituting large-scale publicly funded health insurance. To address a heavily privatized healthcare system leading to a high out-of-pocket payment, the government introduced health insurance schemes, such as the Rashtriya Swasthya Bima Yojana (RSBY) and state-specific programs like the Vajpayee Arogyashree Scheme in Karnataka. In 2018, India launched the Ayushman Bharat-Pradhan Mantri Jan Arogya Yojana (PM-JAY) health insurance scheme with the primary objective to “reduce catastrophic out-of-pocket health expenditure by improving access to quality health care” [6, 7]. PM-JAY is the world’s largest health insurance program in terms of coverage, with about 500 million low-income beneficiaries. It covers medical expenses up to INR 500,000 (about USD 6,750 at the average 2020 exchange rate) annually per family for more than 1,600 health benefits packages across 24 specialties at secondary and tertiary care in participating public and private hospitals [8]. The PM-JAY benefits include cancer treatment among its supported services [9]. PM-JAY is co-funded by the federal and state governments. Participating hospitals receive a fixed “package” payment that is intended to defray costs associated with treatment and post-hospitalization follow-up care for up to 15 days.

Public health insurance schemes in LMICs, like India, often fail to provide adequate coverage for cancer treatments [10]. A major concern is the inadequate public funding for cancer care, with India spending less than $10 per person annually. The private healthcare sector, which is largely unregulated, tends to drive up the cost of cancer care, making it even more challenging for public health insurance programs to meet patient needs. This insufficiency forces patients to rely heavily on out-of-pocket payments, leaving many patients unable to afford essential care. Additionally, the healthcare infrastructure is often unevenly developed geographically, and there is a significant shortage of trained professionals, further complicating the delivery of effective oncology services.

To address these issues, there is a pressing need for better-designed public insurance schemes that offer comprehensive coverage, as well as stronger regulatory measures to control healthcare costs and improve the quality of cancer care. An important concern for insurers like PM-JAY is whether hospitals file claims are appropriate for the care they provided and the quality of this care. In response to these concerns, India’s National Health Authority has deployed several tools to improve hospitals’ capacity to administer the scheme and improve quality, such as training, hospital certification and standard treatment guidelines that are developed in partnership with medical associations and specialty collaborative [8, 11].

These concerns extend to breast cancer treatments, which are covered by about 140 packages in PM-JAY [12]. Breast cancer is the most common cancer globally and also in India, with 211,000 projected cases in 2020 [13]. In 2019–2020, there were 86,000 claims for Breast Cancer treatment under PM-JAY for 29,000 unique beneficiaries [12]. Claims for breast cancer care within PM-JAY vary across states and tend to be concentrated within a small number of specialty hospitals in a state; private hospitals file about half of these claims [12].

Claims for palliative treatment account a large share of oncology claims, with 31% of all oncology volume nationally, or 316,000 of 1,010,000 [14]. The scheme in our study state allows hospitals to file claims for “Palliative Chemotherapy—Unlisted Regimen” which is intended to reimburse chemotherapy given to patients with metastatic cancer. The package also covers other expenses like bed charges, healthcare provider fees, diagnostic charges, and food. As an unlisted regimen, this package can be filed for any palliative chemotherapy regimen and historically received limited oversight. Although it is reimbursed at a relatively low rate of INR 5,000 (USD 67), the package has a disproportionate budget impact because of the high volume of claims. Based on the scheme’s internal data, palliative treatment was by far the most important package within oncology with close to 500,000 million claims (about 57% of all oncology claims) and close to INR 2.5 billion (USD 31.3 million) or about 46% of spending in oncology since the launch of the scheme. Within the overall scheme, it accounts for about 14% of all claims and 3% of all spending.

We conducted a study to assess the appropriateness of package selection according to clinical treatment guidelines for palliative care provided to breast cancer patients, using documentation submitted to an Indian state health insurance scheme. We cannot identify this state by name because of confidentiality reasons and data use agreements.

## Methods

### Data sources

We obtained claims and supporting information from the state health agency for a random sample of 50 female patients with breast cancer who received palliative care at a public hospital (*n* = 25) and a private hospital that participates in the scheme (an “empanelled hospital”; *n* = 25) hospital between May 1, 2019, and April 1, 2020. These records are submitted by hospitals to the agency for reimbursement and include a cover sheet and a variety of other supporting documents, such as consent forms, and lab and diagnostic reports (Table 1). In our study period, the agency recorded a total of 207,306 oncology claims, of which 98,947 were for the “palliative care – unlisted” package, and 8,480 of these were for palliative treatment for patients with breast cancer.

**Table 1:** Available documentation for the index claim

Type of documentation	Count of claims			Percent		
	**Both**	**Public**	**Private**	**Both**	**Public**	**Private**
Invoice (bill)	50	25	25	100%	100%	100%
**Checklist for Unlisted Regimen Palliative Chemotherapy**	50	25	25	100%	100%	100%
Chemotherapy details	**50**	**25**	**25**	**100%**	100%	100%
Pre-authorization form^1^	49	25	24	98%	100%	96%
Consent form	49	24	25	98%	96%	100%
**Biopsy report**	**48**	**25**	**23**	**96%**	100%	92%
Complete blood count report	23	23	0	46%	92%	0%
Biochemistry report	20	20	0	40%	80%	0%
Registration form	19	19	0	38%	76%	0%
Any radiodiagnosis^2^	14	13	1	28%	52%	4%
Ultrasound report	9	8	1	18%	32%	4%
Computer Tomography (CT Scan) report	7	7	0	14%	28%	0%
Counselling form	5	5	0	10%	20%	0%
Fine Needle Aspiration Cytology (FNAC)	5	0	5	10%	0%	20%
Ration card	4	4	0	8%	16%	0%
Chest x-ray	3	3	0	6%	12%	0%

Notes: Sorted in order of “percent both”. Bold-faced rows are required documentation for this package

^1^Includes registration details, consent and counseling of patient and guardian

^2^Radiodiagnosis includes chest x-ray, CT-scan and/or ultrasound

We selected the study hospitals in a two-step procedure. First, we identified the two public and two private hospitals that had the largest volume of *claims for “palliative chemotherapy—unlisted regimen” for all cancers* in our study period. From these hospitals we selected one public and one private hospital with the highest volume of palliative care claims specifically for breast cancer diagnosis (*n* = 686 or 8.1% and *n* = 431 or 5.1% of relevant claims, respectively). We chose this approach to facilitate comparisons between the public and private sector; we discuss possible limitations of our approach below.

We randomly selected 25 patients from each of these two hospitals and obtained detailed records (claim and supporting information) for their index claim. Specifically, we numbered the anonymized palliative care cases from the two hospitals and selected 25 patients each using random number tables. For all 50 patients, we also obtained the claims history (without supporting documentation) between March 19, 2014 and March 15, 2021. As a robustness check, we also obtained detailed records for all claims in this period for five patients, three from the public hospital and two from the private hospital, based on the possibility that additional documentation may have been submitted in other claims for these patients, including from other hospitals. We purposefully selected these patients based on missing documentation during the process of reviewing the index claim. Personal information was redacted after documents were received from the state health agency and before being shared with local members of the study team.

This study did not involve patient and public participation.

### Analysis

We conducted three related analyses for our primary sample of 50 patients: cataloguing the available documentation, assessing appropriateness of package selection, and assessing whether patients received relevant care elsewhere.

First, we assessed the availability of documentation for the index claim by cataloging and tabulating the supporting documentation that was filed with the claim.

**Fig. 1:**
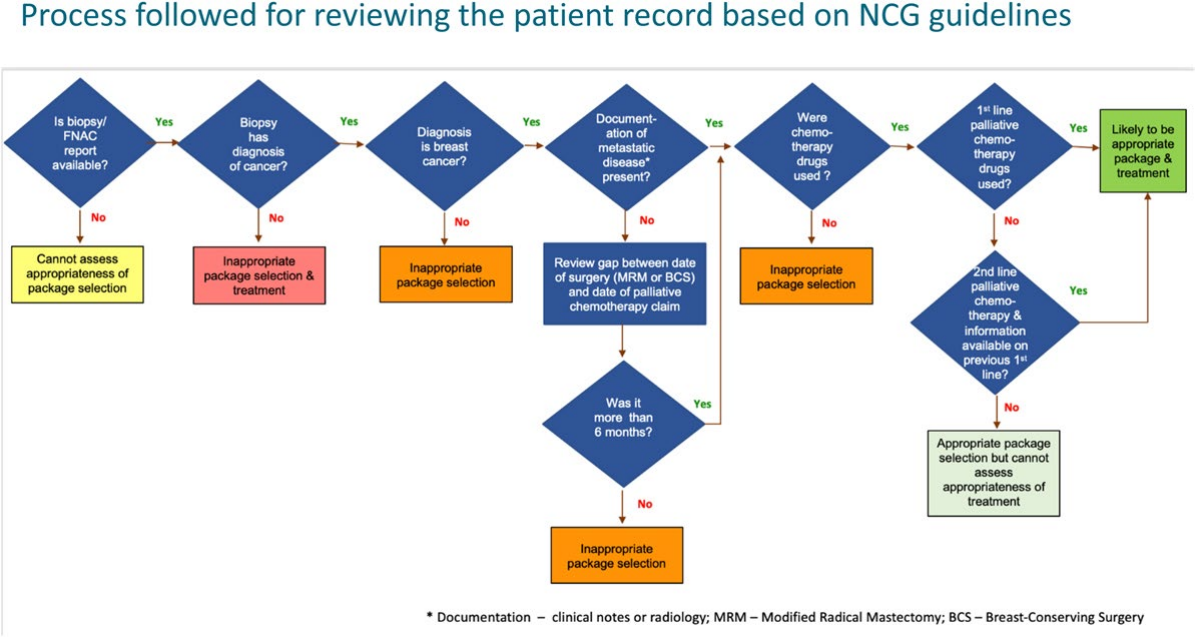
Process followed for reviewing the patient record based on NCG guidelines

Second, we assessed whether the treatment hospitals provided aligned with the package they selected when filing for reimbursement. We assessed appropriateness of the package selection using guidelines developed by the National Cancer Grid [11, 15]. For this purpose, we converted the National Cancer Grid (NCG) guidelines into a flow diagram that describes the sequence of decisions and treatments based on clinical information (Fig. 1) [15]. Specifically, we checked for availability of biopsy or fine needle aspiration cytology (FNAC) and used information from these reports to assess whether a breast cancer diagnosis was established and, if so, whether there was documented metastatic disease. In cases where there was no documentation supporting metastatic disease, we assumed a gap of less than six months between the dates of modified radical mastectomy (MRM) or breast-conserving surgery (BCS) surgery and the palliative chemotherapy claim as being indicative of non-metastatic disease. In patients with established metastatic disease or a gap of at least six months between the dates of MRM or BCS surgery and the palliative chemotherapy claim, we checked whether non-chemotherapy drugs (e.g., hormonal therapy) was provided. If chemotherapy drugs were used, we assessed whether they were first line or second line drugs as per NCG guidelines, if second line drugs were used whether information on prior first line therapy was available. Appendix Table A1 lists the first and second-line drugs that were considered appropriate as per NCG guidelines.

We deemed as inappropriate package selection those cases where there was (i) no documentation of metastatic disease and palliative chemotherapy started within 6 months of breast cancer surgery; (ii) the use of drugs that are not chemotherapy; (iii) when the biopsy showed a diagnosis of a variant breast condition or cancer other than breast. We deemed as appropriate package selection those cases for which there was (i) first-line palliative chemotherapy along with documented metastatic breast cancer or more than 6 months gap between the palliative chemotherapy and breast cancer surgery dates and; (ii) second-line palliative chemotherapy with documented evidence of previous first-line treatment in either documented metastatic breast cancer or a gap of more than 6 months between breast cancer chemotherapy and surgery dates. Appendix Table A1 lists the first and second-line drugs that are considered appropriate per NCG guidelines. We were unable to assess appropriateness if (i) the biopsy report was not available; (ii) second-line palliative chemotherapy was given, and there was no documentation of first-line therapy; (iii) when there was a discrepancy in the regimen listed on the consent form and the clinical notes; (iv) when there was neither documentation of metastasis or information on gap between the chemotherapy and surgery dates. We applied this process to all relevant claims and also tracked the reasons for our determination. Three clinical experts among the authors conducted this assessment and discussed and reconciled any differences. For those claims that had sufficient documentation, we assessed whether or not the reported care was clinically appropriate.

Third, we used the claims history to examine where patients received care under the scheme, by tabulating the share of claims that patients received in the hospital that generated the index claim.

## Results

The availability of supporting documents for the index claim is shown in Table 1. The highest availability was for documents related to claim reimbursements, such as the invoice, checklist (cover sheet), and pre-authorization form. The consent form was available for 49 of the 50 claims. Radiology reports (on chest x-rays, CT scans and/or ultrasound) to corroborate the presence of metastatic disease were available for only 14 (28%) claims. There were some differences in availability of documentation for claims from the public and the private hospital. For example, the public hospital was more likely to have submitted diagnostic details, e.g., ultrasound, x-ray or CT reports.

We could not establish the appropriateness of the package selection for 5 of the total 50 claims (10%, Table 2). For the remaining 45 claims, we found that package selection was inappropriate for 60% (30) and appropriate in 40% (15). For claims with inappropriate package selection, 3 out of 30 (10%) were also deemed to have inappropriate treatment. The remaining 90% (27) of inappropriate package selection claims could not be assessed for treatment appropriateness. For claims that had appropriate package selection (15), 60% (9) were assessed to have appropriate treatment for first line (7) and second line therapy (2) as per NCG guidelines. For the remaining 40% (6), treatment appropriateness could not be assessed. The share of appropriate package selections is larger for the public hospital (*n* = 11 or 48% of the 23 claims for which we could establish appropriateness) than for the private hospital (*n* = 4 or 18% of 22 claims).

**Table 2:** Reasons for the determinations of package selection and treatment appropriateness

	Both		Public	Private
	Count	Percent	Count	Count
**Cannot assess appropriateness of package selection or treatment (*n* = 5)**				
No documentation of metastasis or gap between Palliative care chemotherapy date and breast cancer surgery	3	60%	0	3
Discrepancy in regimen listed on consent form and the clinical notes	2	40%	2	0
**Inappropriate package selection (*n* = 30)**				
**Inappropriate package selection and inappropriate treatment (*n* = 3)**				
**Inappropriate package selection (treatment appropriateness could not be not assessed) (*n* = 27)**				
No documentation of metastatic disease and Palliative care chemotherapy started within 6 months of breast cancer surgery	21	70%	7	14
Biopsy showed diagnosis of cancer other than breast cancer	2	7%	1	1
Drugs that are not chemotherapy used	4	13%	4	0
**Appropriate package selection (*n* = 15)**				
**Likely to be appropriate package selection (treatment appropriateness could not be not assessed) (*n* = 6)**				
Documented breast cancer + documented metastasis or more than 6 months gap between chemotherapy date and surgery + 2nd line Palliative care chemotherapy given but no documentation of 1st line therapy	6	40%	5	1
**Likely to be appropriate package selection and treatment (*n* = 9)**				
Documented breast cancer + documented metastasis or more than 6 months gap between chemotherapy date and surgery + 1st line palliative care chemotherapy	7	47%	4	3
Documented breast cancer + documented metastasis or more than 6 months gap between chemotherapy date and surgery + 2nd line palliative care chemotherapy with documented evidence of previous first line treatment	2	13%	2	0

The reasons for these determinations are listed in Table 2. For packages that are likely to be appropriately selected, all [15] of them documented breast cancer and either metastasis or a gap of six months or more between palliative chemotherapy date and breast cancer surgery. 47% (7) documented the use of first-line palliative care chemotherapy, 13% (2) documented use of second-line palliative care chemotherapy with previous history of first-line therapy and 40% (6) documented second line palliative care chemotherapy but no documented evidence of first line therapy.

The main reason for a determination of inappropriate package selection was the lack of documentation of metastatic disease, evidence that chemotherapy was started within 6 months of breast cancer surgery and the chemotherapy used was an adjuvant regimen (70% of inappropriate package selection). These factors made it unlikely that the patient had metastatic disease requiring palliative chemotherapy. Other reasons were a biopsy that showed diagnoses of cancer other than breast cancer (7%) and the use of drugs other than chemotherapy (13%). We found evidence of care that was clinically inappropriate in 3 out of 30 patients (10%) that had inappropriate package selection.

The reasons why we could not establish appropriateness in 5 cases include missing documentation of metastasis or the gap between the surgery and chemotherapy dates (60%); and discrepancies in the regimen listed on the consent form and the clinical notes (40%).

Our review of all claims for the purposefully selected five patients did not yield any additional insights that would affect these results. Oftentimes, the same documentation available for the index claim had been submitted along with the other claims for the same patient.

Our analysis of the claims histories shows 80% and 96% of claims for our sampled patients from the public and private hospital, respectively, originated from the same hospital (Appendix Figure A1). However, more than 50% of patients of the public hospital had at least one claim from another hospitals, with 3 patients having claims from a total of three hospitals and 10 having claims from two hospitals (Appendix Figure A2). Only 3 patients from the private hospitals had claims from one other hospital. The average number of claims was 14.7 and similar for the public and private hospital at 14.5 and 14.8, respectively.

## Discussion

We examined the appropriateness of hospitals’ selection of the palliative chemotherapy package for care provided to breast cancer patients, using claims submitted by a public and a private sector hospital to a state hospital insurance scheme in India. Among the 45 claims for which we could establish appropriateness of selecting this package, two-thirds (67%) were likely inappropriate selection and one third were appropriate. Claims and reported care from the public hospital were more than twice as likely to be appropriate for this package.

These findings give rise to serious and urgent concerns about the claims processes followed by public and private cancer hospitals, as well as the potential impact on quality of care. The documentation often lacks the information needed to assess the appropriateness of package selection and treatment given. For most claims in our analysis, we needed to infer this information based on clinicians’ review of the indirect parameters, e.g., the presence or absence of metastatic disease by using the gap between modified radical mastectomy and start of chemotherapy. This makes it difficult and inefficient to monitor the quality of claims at scale. Our sample is drawn from hospitals with large volumes for this procedure; smaller hospitals may be even less likely to file appropriately. Regulators should intervene promptly to communicate clinical guidelines and ensure adherence. They could also expand this assessment to random samples of claims from other hospitals and work with hospitals to facilitate improvements. In most cases, packages other than the unlisted palliative chemotherapy package would have been appropriate. This may be due to lack of clarity on the type of documentation and training of the personnel involved in package selection and document submission. Some of the patients who were given adjuvant chemotherapy e.g., with Adriamycin and Cyclophosphamide (AC) under the unlisted package should have been appropriately treated under the adjuvant chemotherapy package available. A large proportion of these alternative packages have a lower reimbursement rate than the palliative care package. Thus, the scheme not only paid for inappropriately selected packages but may also have overpaid hospitals. From another perspective, hospitals may choose the palliative chemotherapy package because alternatives (adjuvant packages) may have been underpriced by the scheme, and may need revision.

The findings are also troubling for the quality of care provided under the scheme. At least some of the care provided under the scheme was not clinically appropriate. The regulator could use the same methodology to also examine quality of care and consider, e.g., public reporting of findings to benchmark performance, incentivizing hospitals to improve, and informing patients.

The state health agency could address these concerns in several ways. First, the agency should conduct additional research to confirm our findings and rule out clerical error in documentation or submission. This may require access to hospital-internal patient records. Second, the agency could consider splitting the catch-all “unlisted” package into more specific packages. Third, the layout of the various forms could be simplified allow for automatic checks, e.g., changing the forms to become machine-readable. This could provide immediate feedback to hospitals and may also reduce the risk of clerical error, which may be acceptable to and even welcome by hospitals. The agency could extend this kind of assessment to other packages by developing audits based on clinical guidelines and applying those to random samples of claims. The implementation should include training of hospital staff for appropriate selection of packages and supportive documentation. Schemes should make use of their claims data to identify, track and address shortfalls for appropriate use of health benefits packages. They should also use the submitted documents to assess the quality of clinical care. More broadly, schemes could also more effectively engage patients, e.g., through collecting feedback and proactively informing patients about appropriate care. These clarifications and trainings could be implemented within a relatively short time frame, e.g., within 6 and 12 months, respectively. Implementing some of the other proposed changes like, use of evidence-based clinical pathways, robust information systems, and strong monitoring and regulatory mechanisms are more medium to long term. Recent efforts by the national insurers and professional associations to standardize care using clinical guidelines and investments by the Government of India in digital health systems, provide opportunities that can be leveraged [16].

The agency has already implemented a related suggestion: to revise its requirements for documentation. In particular, the agency revised the cover sheet to capture all data required to assess package appropriateness (rather than having this information scattered across different documents) and instituted mandatory checkboxes to clarify if a cancer was metastatic or non-metastatic, as well as a clinical note signed by the responsible physician. Radiological confirmation of metastatic disease is to be submitted as evidence if available. The agency also requires additional documentation, e.g., for metastatic disease the clinical note includes details on whether therapy is first line/ second line (with names of previous lines of treatment) and after 3 cycles of chemotherapy it should be documented whether the patient has responded. The revised requirements are unlikely to introduce additional administrative burden for hospitals and, indeed, may have clarified the process and reduced the risk of errors, claim denials and potential fraud.

Our study provides an example of using routine claims data for evaluating the appropriateness of package selection as well as the quality of hospital care. Appropriate filing is crucial for insurance programs’ financial sustainability and evolution, e.g., by adjusting payment rates or modifying package definitions. Routine data also has potential to monitor and improve the quality of care using actual clinical data when available. Our study shows how to rigorously assess administrative and clinical adherence by codifying treatment guidelines, drawing random samples and using documentation that is filed as part of the routine claims process. Analyses like ours can also complement the use of claims for audit and tracking purposes [17], as well as facilitate quality measurement as it is commonly done in high-income health systems (e.g., [18]). More broadly, our analysis highlights challenges in implementing and monitoring essential health benefit packages in low and middle-income countries [19, 20].

Future research could investigate the impact of revised documentation requirements on hospital practices and assessing the long-term effects of policy changes on the appropriateness of package selection. Moreover, qualitative research could investigate the perspectives of healthcare providers and patients regarding package selection and the quality of care. Such research could also examine additional opportunities and implementation challenges to improving documentation and care.

### Limitations

Our analysis has several limitations. First, patients may have received care that is not described in the documents that hospitals file to support a claim. Investigating this possibility would require access to hospital-internal records, such as patient files. Second, patients may have received additional care outside of the scheme or for which empaneled hospitals did not submit claims to the state health agency. Third, our sample of hospitals and patients may not be representative. In particular, our data and findings pertain to specific group of hospitals with relatively large claims volumes for this package; patterns and adherence may differ across other hospitals in or outside of the insurance scheme. The hospitals may also differ from other hospitals in the state and India, e.g., in terms of governance and management, and the state may also be different from other states, e.g., in leadership and governance. The patients at these hospitals may also be systematically different from the broader population of breast cancer patients in the scheme. Both considerations could affect the generalizability of our findings. Finally, our claims data are at risk of coding errors or (as per our results) variations in reporting practices. As a result, the documentation we reviewed may not always and fully represent the care that patients received. The implications for our findings related to quality of care are unclear, as the correlation between more complete documentation and appropriateness is uncertain.

## Conclusion

We found that few claims for the palliative care package were appropriate for the palliative chemotherapy package and majority of the claims were incorrectly filed. We also found some cases where the reported care was clinically inappropriate based on the documentation submitted to the insurance scheme. There is an urgent need to further investigate the scale, scope and reasons for non-adherence to administrative and clinical guidelines and to develop and deploy interventions to significantly improve adherence. Specific interventions could include increasing the number of specific packages with explicit indications regarding the line and type of therapy based on clinical guidelines instead of having ambiguous, non-specific packages; instituting systems that clearly lay out the required documentation and indicators to monitor compliance to administrative and clinical guidelines; making documents machine-readable; directly linking the packages with electronic clinical records to avoid duplication and associated potential errors; and training of healthcare providers, care coordinators and administrative staff on clinical guidelines, documentation and appropriate coding.

Our study highlights an important aspect of the implementation of essential health benefits plan with implications for quality of care, optimal resource utilization and rationalization of packages included in universal health coverage in low resource settings.

## Supplementary Information

Supplementary Material 1.

## Data Availability

The data used in this study are the property of the state health insurance scheme and are not publicly available.
