# Supplementary material for: Health benefits package selection in oncology: a clinical audit of palliative chemotherapy for breast cancer in an Indian health insurance scheme
Source: BMC Health Serv Res. 2024 Dec 18;24:1591. doi: 10.1186/s12913-024-12018-4 (PMC11654404; doi:10.1186/s12913-024-12018-4)
Supplement: Supplementary file 1 — Supplementary Material 1. [file 12913_2024_12018_MOESM1_ESM.pdf]

Supplemental Material

Figure A1. Distribution of claims based on claims history

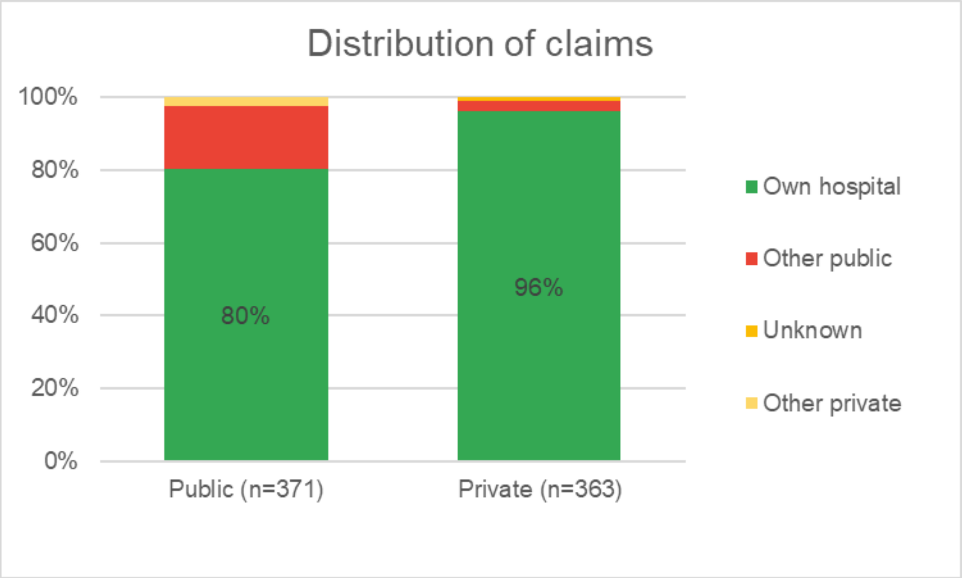

Note: based on claims history between March 19, 2014 and March 15, 2021.

Figure A2. Number of hospitals per patient based on claims history

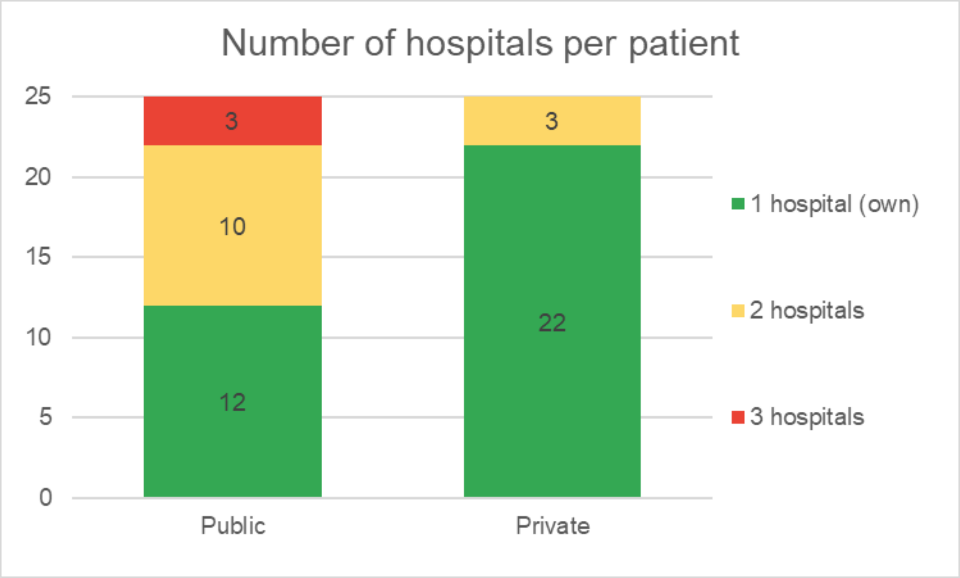

Note: based on claims history between March 19, 2014 and March 15, 2021.

Table A1: Drugs for metastatic breast cancer per NCG guidelines

ER + PR + Her 2		HER 2 positive	TNBC
First Line Therapy			
Low tumor burden:<br>Letrozole/ Anastrazole/ Exemestane / Tamoxifen<br>Plus Ribociclib or Palbociclib	Visceral crisis:<br>Taxane	Taxane plus Trastuzumab	Taxane
	Adriamycin Cylclophosphamide (AC) / Epirubicin Cylclophosphamide (EC)		Adriamycin Cyclophosphamide (AC) / Epirubicin Cyclophosphamide (EC)
			Taxane plus platinum
Second Line Therapy			
Fulvestrant or Everolimus and Exemestane	Capecitabine	Lapatinib plus Capecitabine	Capecitabine
Also in PIK 3 CA mutant - Alpelisib plus Fulvestrant	Gemcitabine Carboplatin	TDM1	Gemcitabine platinum
If premenopausal then Ovarian suppression - Avoid Tamoxifen with Ribociclib		Hormone therapy as maintenance of ER/ PR positive	Eribulin
		-	Olaparib or Rucaparib for any patient with Germline or somatic mutation
